# Exploring agro-ecological significance, knowledge gaps, and research priorities in arbuscular mycorrhizal fungi

**DOI:** 10.3389/fmicb.2024.1491861

**Published:** 2024-11-01

**Authors:** Lenganji Lackson Mwampashi, Aneth Japhet Magubika, Job Frank Ringo, Dickson J. Theonest, George Muhamba Tryphone, Luseko Amos Chilagane, Eliakira Kisetu Nassary

**Affiliations:** ^1^Department of Crop Science and Horticulture, College of Agriculture, Sokoine University of Agriculture, Morogoro, Tanzania; ^2^Department of Soil and Geological Sciences, College of Agriculture, Sokoine University of Agriculture, Morogoro, Tanzania

**Keywords:** molecular imaging, nutrient uptake, soil health, sustainable agriculture, symbiotic relationships

## Abstract

This systematic review examines the global agricultural relevance and practical environmental implications of arbuscular mycorrhizal fungi (AMF) within the phylum Glomeromycota. Following PRISMA guidelines, ensuring a comprehensive and unbiased literature review, a literature search was conducted, focusing on the functional roles of AMF in enhancing crop productivity, nutrient uptake, and soil health. Key findings reveal that AMF contribute significantly to sustainable agriculture by reducing the need for chemical fertilizers and increasing plant resilience to environmental stressors like drought, salinity, or pest resistance. The review highlights the importance of AMF in forming symbiotic relationships with plants, which enhance nutrient absorption and improve soil structure, showcasing long-term benefits such as reduced erosion or improved water retention. However, the current literature lacks in-depth exploration of the taxonomy and evolutionary aspects of AMF, as well as the specific functional roles they play in different agricultural contexts, e.g., understanding evolution could enhance strain selection for specific crops. This review identifies several urgent research gaps, including a need for a more refined understanding of AMF community dynamics under varying land management practices. For example, there are gaps in and a critical evaluation of advanced molecular techniques. Such techniques are essential for studying these interactions. Addressing these gaps will enhance the integration of AMF into sustainable agricultural systems and improve ecosystem management practices across different geographical regions. Future research should prioritize developing precise molecular imaging techniques and optimizing AMF applications for different crops and soil types to maximize their ecological and agricultural benefits. This could be practical through interdisciplinary collaboration (e.g., involving molecular biologists, agronomists, etc.). In conclusion, this review advances the practical application of AMF in agriculture and its contribution to biodiversity conservation in agroecosystems. Integrating these findings into policy frameworks could encourage sustainable farming practices, promote the adoption of AMF inoculants, and foster incentives for environmentally friendly land management strategies.

**Systematic review registration:**

https://www.bmj.com/content/372/bmj.n71.

## 1 Introduction

The taxonomy of fungi, particularly those forming arbuscular mycorrhizae in the roots of terrestrial plants, has historically been challenging due to difficulties in isolating spores from soil and maintaining live cultures (Wilkes, 2021; Khaliq et al., 2022), this could be due to the complex soil environment, where spores occur in low abundance and are often entangled with other microorganisms, and because arbuscular mycorrhizal fungi are obligate symbionts, requiring a host plant to complete their life cycle. Early taxonomic efforts based largely on morphological characteristics, grouped fungi with similar structures into broad categories (e.g., spore size, hyphal structure). For instance, the Endogonaceae family, initially associated with vesicular-arbuscular mycorrhizae (VAM), was once thought to belong to the order Mucorales due to its production of non-septate hyphae and zygospores (Morton et al., 1990), a view later revised through molecular studies. However, subsequent studies revealed distinct molecular and morphological features, prompting reclassification (e.g., using gene sequencing) into separate orders and even phyla (Desirò et al., 2017; Yamamoto et al., 2015).

Fungi now classified within Mucoromycotina and Glomeromycota were once grouped under Zygomycota due to shared features like non-septate hyphae and zygospore formation. As molecular techniques, such as DNA sequencing, became more prevalent, these groups were found to have significant genetic differences, justifying their reclassification into separate phyla. Molecular markers, such as ribosomal RNA genes, which are essential in fungal taxonomy due to their conserved nature and variable regions that help distinguish species and trace evolutionary relationships have been pivotal in these taxonomic revisions, highlighting both the diversity and the evolutionary distinctions between these groups (Schüβler et al., 2001; Redecker et al., 2006).

Recent advancements in molecular biology have allowed for a more refined classification of fungi that form arbuscular mycorrhizae. For example, the development of high-throughput sequencing or genomic analysis has revealed genetic signatures unique to AMF, leading to the establishment of the orders Archaeosporales, Diversisporales, Glomerales, and Paraglomerales within the phylum Glomeromycota, each with distinct ecological roles, such as specialization in different habitats or adaptation to varied environmental conditions (Schwarzott et al., 2001; Spatafora et al., 2016; Stürmer and Kemmelmeier, 2020). This molecular approach has provided deeper insights into the evolutionary history of these fungi and clarified their relationships with other fungal groups, enhancing our understanding of their functional roles in ecosystems (e.g., phosphorus uptake, soil aggregation).

Understanding the taxonomy and evolutionary history of AMF is not merely an academic exercise; it has significant practical implications for agriculture and ecosystem management (e.g., improving phosphorus uptake in crops). AMF play a crucial role in plant nutrient uptake, particularly phosphorus, which is often a limiting factor in soil fertility by stabilizing soil aggregates. By forming symbiotic relationships with plant roots, AMF enhance nutrient absorption, promote soil structure, and increase plant resilience to environmental stressors. This makes them invaluable for sustainable agriculture, as through reduction in fertilizer use by a percentage or specific crop improvement (Kumar et al., 2023; Verbruggen and Kiers, 2010). Despite these benefits, there remain significant knowledge gaps in an understanding of AMF. For instance, the diversity of AMF species and their specific interactions with different plant hosts and soil types are not well understood (e.g., to match AMF strains with specific crops for better outcomes). This lack of knowledge hinders the development of targeted strategies for using AMF in agriculture and soil management. Moreover, the evolutionary adaptations that enable AMF to thrive in various ecological niches are not fully understood, limiting the ability to predict their responses to changing environmental conditions (Brundrett, 2002; Li et al., 2021).

This review aims to address these gaps by synthesizing current research on the taxonomy, evolutionary history, and ecological significance of AMF. By focusing on the molecular and morphological features that distinguish AMF from other fungal groups, this review seeks to provide a clearer understanding of their role in ecosystems and their potential applications in sustainable agriculture. Additionally, this review will highlight specific agricultural challenges, such as phosphate limitation, where AMF could provide significant benefits, offering new insights into their practical use in modern farming practices. By narrowing the focus to these areas, this review aims to advance the practical application of AMF in agriculture and contribute to biodiversity conservation in agroecosystems, particularly by supporting plant diversity through enhanced nutrient acquisition and symbiotic relationships.

Based on the existing literature base, this systematic review sought to consolidate existing knowledge on AMF and pinpoint key areas for future research. To achieve this, three primary objectives were established: first, to examine the taxonomic, evolutionary, and ecological dimensions of AMF within the phylum Glomeromycota. It will focus on both historical and contemporary challenges in AMF taxonomy, evolutionary development, and ecological functions. Second, this review aims to evaluate the practical implications of AMF’s roles across various ecosystems, with a particular emphasis on agriculture. It highlights how AMF contribute to soil health, nutrient uptake, and plant resilience, thus underscoring their significance in agricultural practices. Third, to uncover specific knowledge gaps in AMF research and recommend future research directions by assessing the limitations of current methodologies (e.g., molecular, ecological) and enhancing understanding of AMF diversity and functionality across different environmental and agricultural contexts.

## 2 Literature search

**Figure 1:**
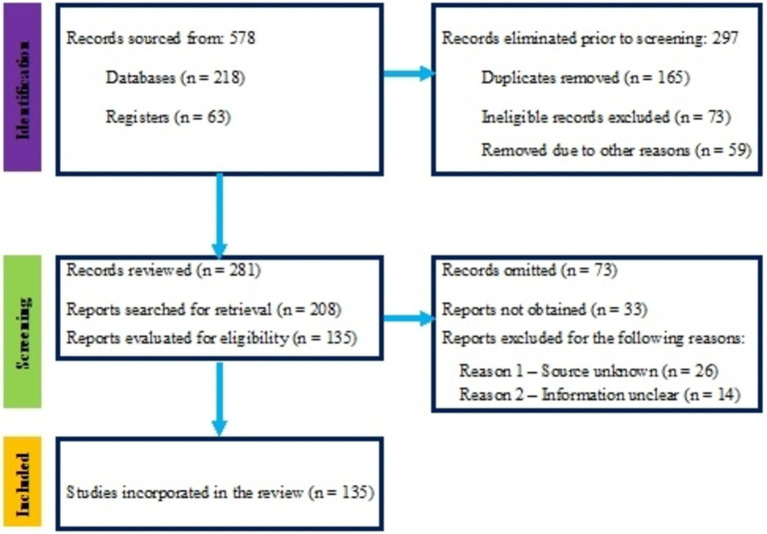
Number of studies identified for eligibility in the presented study.

The review followed the Preferred Reporting Items for Systematic Reviews and Meta-Analyses (PRISMA) guidelines (Table 1; Figure 1) to ensure a transparent and comprehensive process as compiled by Page et al. (2021). PRISMA is important for systematic reviews because it provides a standardized framework that enhances the clarity, rigor, and reproducibility of the review process, thereby increasing the credibility and reliability of the findings presented. The PRISMA framework was applied throughout the literature search and selection phases to identify and evaluate relevant studies. Specifically, this review emphasized defining clear inclusion and exclusion criteria, systematically conducting the literature search across multiple databases, and documenting the selection process to ensure transparency and replicability in identifying studies that met the criteria for inclusion. The literature search was conducted using multiple databases, including MEDLINE, Cochrane Library, Scopus, and Web of Science, known for their extensive coverage of health-related, ecological, and evolutionary studies. The Cochrane Library is recognized for high-quality systematic reviews in health sciences, while Scopus offers broad interdisciplinary coverage, especially in social and life sciences. Web of Science excels in indexing high-impact journals and citation analysis. Grey literature sources like Google Scholar were also used to ensure comprehensive coverage. Including grey literature is significant because it often contains valuable research findings, such as thesis, reports, and conference proceedings, that may not be published in peer-reviewed journals. This approach enhances the depth of the review by capturing emerging studies and insights relevant to understanding complex ecological and agricultural dynamics. Search terms included combinations such as “arbuscular mycorrhizal fungi + taxonomic,” “arbuscular mycorrhizal fungi + evolutionary,” “arbuscular mycorrhizal fungi + ecology,” and “Glomeromycota + taxonomy,” among others. These terms were chosen based on prior literature and specific research questions to ensure that the search captured a comprehensive range of relevant studies addressing the taxonomic, evolutionary, and ecological dimensions of arbuscular mycorrhizal fungi. Boolean operators (AND, OR) were utilized to refine the search results. Using “AND” narrowed the results by ensuring that all specified terms appeared in the studies, while “OR” broadened the search to include any of the listed terms. This combination enabled a targeted yet comprehensive retrieval of relevant literature on arbuscular mycorrhizal fungi.

**Table 1:** Aligning the study search strategy with the PRISMA flowchart items with the checklist of items by Page et al. (2021).

Stage	Process	PRISMA Checklist Item(s)	Details
Identification	Database search and grey literature review	Item 1: Identify information sources	Conducted comprehensive searches in MEDLINE, Cochrane Library, Scopus, Web of Science, and Google Scholar to gather relevant literature on AMF taxonomy, evolution, and ecology.
Screening	Removal of duplicates and initial screening	Items 4 and 7: Study selection process	Duplicates were removed. Titles and abstracts of remaining studies were screened for relevance to AMF taxonomy, evolutionary aspects, and practical implications in agriculture.
Eligibility	Detailed assessment of full-text articles	Items 6 and 8: Eligibility criteria and reasons for exclusion	Full-text articles were reviewed against inclusion criteria, which focused on AMF’s taxonomic, evolutionary, and ecological aspects, and their practical implications. Studies not meeting criteria were excluded, with reasons documented.
Included studies	Qualitative synthesis of relevant studies	Item 12: Data synthesis methods	Selected studies were synthesized to assess taxonomic classifications, evolutionary trajectories, ecological roles, and practical applications of AMF.
Synthesis	Narrative and comparative analysis of findings	Items 13 and 15: Synthesis of results and additional analyses	A narrative synthesis was conducted to integrate findings on AMF’s ecological significance, practical implications, and research gaps. Comparative analysis was used to highlight patterns and inconsistencies.
Excluded studies	Documentation and reasons for exclusion	Item 7: Study selection process	Detailed records of studies excluded after full-text review, including reasons for exclusion, were maintained to ensure transparency in the selection process.

Studies were included based on specific criteria: they had to be peer-reviewed and address taxonomic challenges, evolutionary history, or ecological roles of AMF within the phylum Glomeromycota. The requirement for peer-reviewed studies is crucial as it ensures the quality and reliability of the research, as these studies have undergone rigorous evaluation by experts in the field, thereby enhancing the credibility of the findings included in this review. Articles discussing AMF’s practical applications in agriculture or providing insights into their symbiotic relationships were also considered. The AMF’s ecological functions can inform sustainable agricultural practices, bridging the gap between theoretical knowledge and real-world implementation to enhance soil health and improve crop productivity. Studies not focused on AMF or irrelevant to the review’s objectives were excluded. The screening and selection process involved removing duplicates, reviewing titles and abstracts for relevance, and obtaining full-text articles for those that met the initial criteria. These articles were then evaluated against the inclusion and exclusion criteria. Studies that did not provide clear data on AMF taxonomy, evolution, or ecology were excluded, as were those that did not contribute novel data or perspectives.

Weighting and bias-correction methods were integral to the analysis of included studies in this systematic review. Although formal statistical weighting or bias-correction techniques were not utilized, weighting in this context refers to the process of evaluating the relative importance or contribution of each study based on its quality and relevance to the research questions. This is essential for systematic reviews as it helps ensure that more reliable studies have a greater impact on the overall findings, thus enhancing the credibility and validity of the conclusions drawn from the review. Sensitivity analyses were conducted to examine how variations in study quality might affect the overall findings. Quality and relevance were assessed using criteria such as the clarity of research objectives, robustness of methodology, appropriateness of statistical analyses, and relevance to the taxonomic, evolutionary, and ecological dimensions of arbuscular mycorrhizal fungi. Geographic distribution was carefully considered to provide a comprehensive view of AMF’s ecological impact. Studies from various regions, including those from the North Caucasus (Yurkov et al., 2023) and karst ecosystems (Xiao et al., 2021), were included to capture a broad spectrum of AMF diversity and functions. Geographic distribution was categorized by region and ecosystem type, allowing for an analysis of how different environmental contexts influence the diversity and ecological roles of AMF across various habitats.

This approach aimed to mitigate potential geographic biases by highlighting areas with limited research and suggesting future studies in these underrepresented regions to improve the understanding of AMF’s global ecological roles. The North Caucasus and karst ecosystems were selected due to their unique characteristics, such as diverse climatic conditions and varied soil types, which create distinct ecological niches for AMF. A narrative synthesis was conducted to summarize the findings, identify patterns, and highlight gaps in the literature. Based on this synthesis, future studies will suggest specific research questions and methodologies to address gaps, guiding researchers in underrepresented areas and enhancing the understanding of AMF’s ecological roles.

## 3 Synthesis of the findings

### 3.1 Evolutionary history of AMF

Fungi have a rich evolutionary history that spans millions of years. The earliest known fungal fossils, dating back to the Ordovician period around 460 to 455 million years ago, offer glimpses into their early existence (Redecker et al., 2000). These fossils predate the emergence of vascular land plants, which appeared around 425 million years ago (Carris et al., 2012). Molecular data suggest that fungi might have arisen over a billion years ago, challenging traditional views based solely on fossil evidence (Bonneville et al., 2020). Late Cretaceous period mushrooms preserved in amber, approximately 94 million years ago, show that modern-looking mushroom-forming fungi coexisted with dinosaurs (Carris et al., 2012; Hibbett et al., 2003). While fossils offer valuable insights, they present a fragmentary view of fungal evolution, with molecular data helping to fill in the gaps and extend the understanding of fungal history (Bonneville et al., 2019).

The AMF play a critical role in terrestrial ecosystems by forming mutualistic relationships with most land plants. These fungi belong to the phylum Glomeromycota and include a diverse array of families such as Acaulosporaceae, Glomeraceae, and Gigasporaceae. The symbiosis between AMF and plants is ancient, dating back at least 450 million years, and has been essential for the colonization of terrestrial habitats (Lanfranco et al., 2016; Vigneron et al., 2018). As obligate biotrophs, AMF rely on their host plants to complete their life cycles, and their evolution is closely linked to the success and expansion of land plants (Kumar et al., 2023). Understanding the evolutionary history of AMF is crucial for harnessing their benefits in modern agricultural and conservation practices. For example, AMF can enhance soil health, improve plant nutrient uptake, and increase crop resilience to environmental stressors, which is directly informed by knowledge of their evolutionary adaptations (Vigneron et al., 2018). These adaptations, including specialized structures for nutrient exchange and symbiotic relationships with various plant species, enable AMF to facilitate nutrient acquisition and enhance plant stress tolerance. Understanding these traits allows for their strategic application in agriculture, promoting sustainable farming and improving crop productivity in diverse environments.

The classification of AMF has historically been fraught with challenges due to their morphological similarities with other fungi. Early taxonomic approaches often grouped AMF with morphologically similar fungi, leading to inaccurate classifications. For example, the Endogonaceae family was previously categorized under the Mucorales order because of similarities in their non-septate hyphae and zygospores (Morton et al., 1990). Such broad classifications based on morphology alone obscured the true diversity and relationships within the AMF group (Wilkes, 2021). This historical confusion has had implications for the functional application of AMF, as misidentification could lead to suboptimal use of AMF in agricultural or ecological settings. This historical confusion has had significant implications for the functional application of AMF, as misidentification can lead to suboptimal use in agricultural or ecological settings, ultimately hindering the potential benefits that these fungi can provide.

Traditional taxonomic methods, which heavily relied on morphological traits, were limited in their ability to capture the full diversity of AMF. Challenges in isolating and culturing AMF spores contributed to incomplete and sometimes inaccurate classifications (Khaliq et al., 2022). These limitations resulted in many AMF being misclassified under broader fungal categories, which hindered understanding of their true diversity and evolutionary relationships (Desirò et al., 2017; Yamamoto et al., 2015). Accurate taxonomy is essential for identifying AMF species with specific functional traits, such as those beneficial for soil health or plant growth. For instance, correctly identifying AMF species that enhance phosphorus availability can lead to targeted applications in agriculture, improving crop yields and reducing the need for chemical fertilizers, thereby promoting sustainable farming practices.

Recent advances in molecular techniques have significantly improved AMF taxonomy. Molecular markers, such as ribosomal RNA genes, have enabled the reclassification of AMF into more accurate groups, revealing new orders and phyla. For instance, the discovery of the order Archaeosporales through molecular analysis has provided insights into AMF that form symbiotic relationships with a wider range of plant species, including those in nutrient-poor environments. The phylum Glomeromycota has been subdivided into distinct orders, including Archaeosporales, Diversisporales, Glomerales, and Paraglomerales, based on molecular data (Schüβler et al., 2001; Redecker et al., 2006). These advancements have refined understanding of AMF taxonomy and highlight the importance of ongoing molecular research to further clarify fungal classifications (Spatafora et al., 2016; Stürmer and Kemmelmeier, 2020). In practical terms, accurate classification allows for the targeted application of AMF in agriculture and conservation, enabling the selection of specific AMF species or strains that can enhance soil fertility, improves plant health, and support ecosystem restoration efforts.

Fungi engage in a variety of mycorrhizal associations beyond AMF, including ectomycorrhizas (ECM) found in specific plant families such as pines and oaks, and vesicular-arbuscular mycorrhizas (VAM), which are widespread among many plant species (Brundrett, 2002). AMF penetrate plant root cells and form arbuscules to facilitate nutrient exchange, and are prevalent among herbaceous plants and crops (Genre et al., 2005; Wahab et al., 2023), whereas ECM primarily form a protective sheath around root surfaces and typically do not penetrate root cells. The biodiversity of mycorrhizal fungi is vast, influenced by factors such as soil composition, climate, host plant diversity, and ecological interactions (Janowski and Leski, 2022; Martin, 2024). Understanding this biodiversity is crucial for optimizing the functional applications of mycorrhizal fungi. For instance, knowing the specific mycorrhizal types and their ecological roles can guide the use of AMF in enhancing agricultural productivity, restoring degraded ecosystems, and promoting sustainable land management practices.

### 3.2 The practical implications of AMF’s roles across various agricultural ecosystems

The AMF are vital for soil health and plant productivity in both natural and agricultural ecosystems. These fungi form symbiotic relationships with plant roots, extending their hyphal networks into the soil to enhance water and nutrient uptake (e.g., improved yields, increased soil carbon storage). This interaction improves soil structure, supports nutrient cycling, and contributes to overall plant health, which is crucial for sustainable agricultural practices (Verbruggen and Kiers, 2010; Powell and Rillig, 2018). The extensive networks created by AMF also play a significant role in stabilizing soil and sequestering carbon, thereby aiding in soil fertility and climate change mitigation (Verbruggen et al., 2015; Wang et al., 2016).

AMF functionality in agricultural ecosystems is influenced by several environmental factors and land management practices. Factors such as soil moisture, temperature, pH, and nutrient availability are key determinants of AMF activity and root colonization. Management practices, including tillage and agrochemical use, can significantly impact AMF networks. For example, tillage disrupts hyphal networks, reducing the fungi’s ability to colonize plant roots effectively, while excessive fertilizers and pesticides can negatively affect AMF populations and their interactions with other soil microorganisms (Powell and Rillig, 2018; Wahab et al., 2023).

Crop diversity significantly affects AMF communities, enhancing their resilience and functionality. Diverse cropping systems like cover cropping and crop rotation promote varied AMF networks that facilitate nutrient exchange and support ecosystem functionality by fostering species coexistence and enhancing nutrient cycling. This diversity is crucial for maintaining ecosystem stability and productivity, especially under varying environmental conditions (Ferlian et al., 2018; Lugo et al., 2023). AMF also interact with a wide range of soil microorganisms, forming complex microbial communities that regulate nutrient cycling and soil fertility. These interactions are essential for maintaining soil health, promoting carbon sequestration, and enhancing plant-microbe relationships in agroecosystems (Frąc et al., 2018).

The adaptability of AMF to different environmental conditions and their ability to form diverse communities are crucial for their role in sustainable agriculture (Table 2). Research indicates that AMF community dynamics change with ecological succession, with shifts towards more specialized fungi and different interaction patterns over time (Bennett et al., 2013). Factors such as environmental changes, seasonal variations, and land-use practices also influence AMF community composition and diversity, highlighting their ability to adapt to ecological shifts. For instance, long-term agricultural practices, such as continuous cropping or monoculture, can reduce AMF diversity, emphasizing the need for sustainable land management practices that foster diverse AMF communities (Higo et al., 2019).

**Table 2:** Quantitative data on the use of AMF in improving soil health, crop production, and carbon sequestration in different parts of the world.

Location	Crop	AMF species	Parameter	No AMF	With AMF	Improvement (%)	References
India	Wheat (*Triticum aestivum*)	*Rhizophagus intraradices*	Grain yield (kg/ha)	2,500	3,200	28%	Sharma et al. (2011)
Brazil	Soybean (*Glycine max*)	*Claroideoglomus etunicatum*	Root biomass (g/plant)	2.5	3.7	48%	Vieira et al. (2020)
Spain	Olive (*Olea europaea*)	*Funneliformis mosseae*	Soil aggregate stability (%)	45	65	44%	Montes-Borrego et al. (2014)
China	Rice (*Oryza sativa*)	*Glomus mosseae*	Carbon sequestration (Mg C/ha)	1.2	1.8	50%	Suriyagoda (2022).
Kenya	Maize (*Zea mays*)	*Gigaspora margarita*	Plant height (cm)	110	140	27%	Malembaka et al. (2021)
USA	Tomato (*Solanum lycopersicum*)	*Rhizophagus clarus*	Phosphorus uptake (mg/plant)	15	25	67%	Higo et al. (2020)
Scotland	Barley (*Hordeum vulgare*)	*Funneliformis caledonium*	Soil microbial biomass (mg/kg)	400	560	40%	Mwafulirwa et al. (2021)

**Figure 2:**
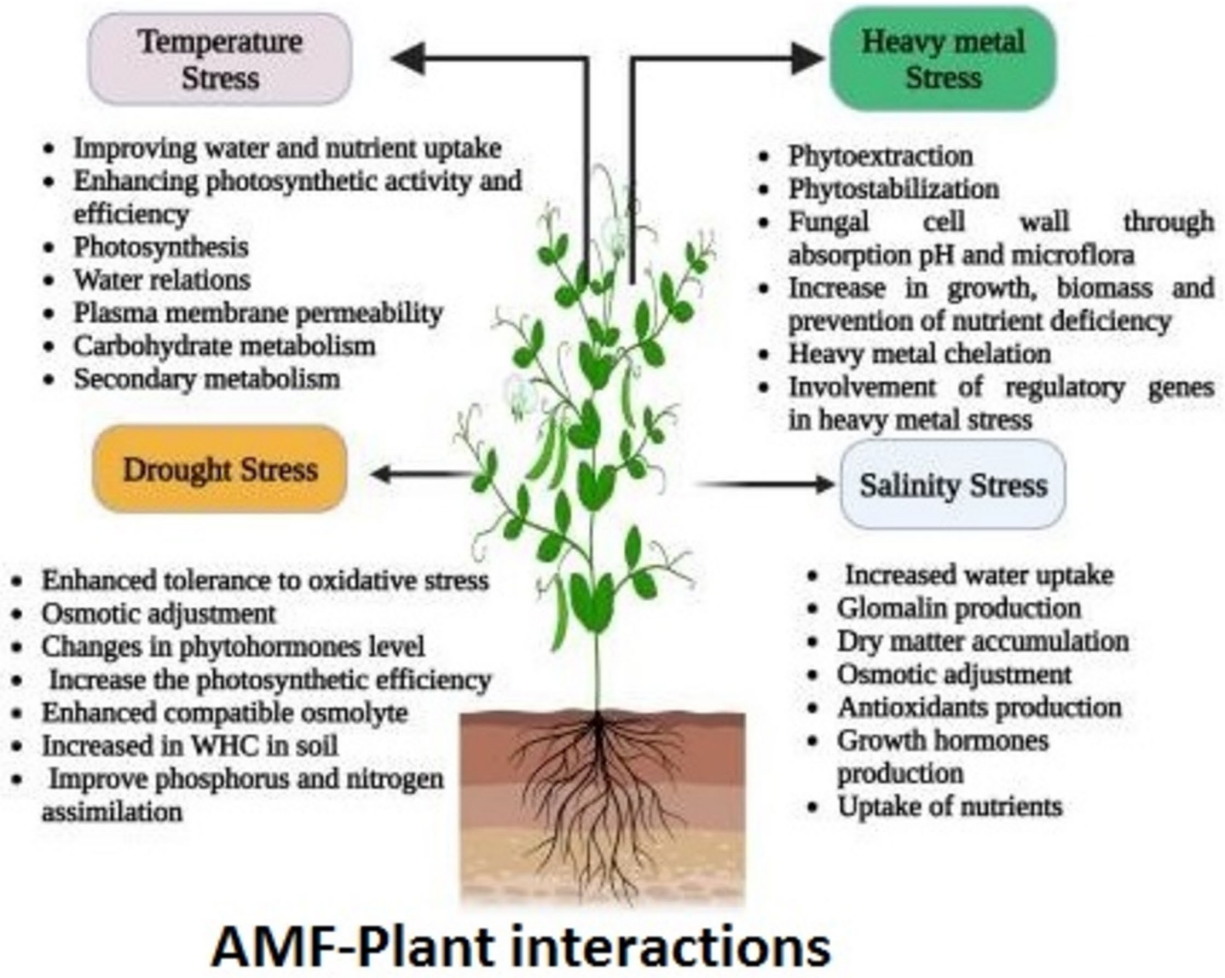
Multi-functionality of AMF in plant growing systems. A visual representation reproduced from Figure 3 of ‘Schematic representation of the different mechanisms imparting abiotic stress tolerance in plants by arbuscular mycorrhiza fungi (AMF)’ by Wahab et al. (2023) under CC BY-NC-ND 4.0 DEED.

Beyond nutrient acquisition, AMF play multiple roles in promoting soil health and ecosystem functionality (see Figure 2), particularly in nutrient-poor environments where they enhance plant growth and stress tolerance (Enebe and Erasmus, 2023; Hu et al., 2024). Their main roles include improving nutrient uptake efficiency, as AMF enhance the availability of essential nutrients to plants, particularly phosphorus; reducing dependency on chemical fertilizers by decreasing the need for chemical inputs; enhancing soil health through improved soil structure, which increases water retention and aeration; promoting stress tolerance, helping plants withstand environmental stressors such as drought and salinity; and supporting ecosystem functionality by playing a crucial role in nutrient cycling and overall ecosystem resilience. This makes AMF an integral component of sustainable agricultural practices aimed at enhancing soil fertility, increasing crop yields, and promoting environmental sustainability (Neuenkamp et al., 2019).

Research into indigenous AMF species has demonstrated their potential as effective biofertilizers, improving crop growth and yield across various soil conditions, including rice cultivation (Kalamulla et al., 2021). Findings suggest the importance of leveraging local AMF species that are well-adapted to specific soil and climatic conditions, enhancing their effectiveness in promoting plant growth and soil health. Utilizing native species over introduced ones offers significant benefits, particularly concerning ecosystem resilience and adaptability, as indigenous AMF are better suited to local environments, enabling them to thrive under varying conditions. This adaptability contributes to stronger plant health and improved nutrient cycling, ultimately fostering greater ecosystem stability. Additionally, studies in diverse ecosystems, such as forests and semi-arid regions, reveal the adaptability of AMF communities to different soil properties and seasonal changes, demonstrating their ecological versatility and importance in various environments (Vieira et al., 2020; Djotan et al., 2024).

Understanding the ecological dynamics of AMF is crucial for their role in sustainable agriculture and ecosystem management. Insights into the interactions between AMF, plants, microorganisms, and environmental factors can inform the development of innovative agricultural practices that harness the benefits of AMF. This knowledge is essential for promoting soil health, enhancing crop productivity, and supporting biodiversity, which are vital for sustainable food production and environmental conservation. Integrating AMF into agricultural systems aligns with global sustainability goals, such as improving food security, mitigating climate change, and promoting biodiversity conservation (Enebe and Erasmus, 2023; Hu et al., 2024). Specifically, AMF can help mitigate climate change by enhancing soil carbon sequestration and improving water-use efficiency in crops, making them more resilient to extreme weather conditions. Successful initiatives, such as incorporating indigenous AMF species in agroforestry systems, have demonstrated significant improvements in crop resilience and soil health, addressing both food security and environmental challenges. Ongoing research continues to unveil the complexities of AMF interactions and their ecological roles, underscoring their potential contribution to sustainable agriculture and ecosystem management. Integrating AMF into agricultural systems can promote soil health, increase crop yields, and support environmental sustainability, contributing to a more sustainable and resilient global food system.

The integration of molecular and ecological insights into AMF has profound implications for agricultural productivity, ecosystem management, biotechnology, and soil health monitoring (Table 3). Advances in molecular techniques and ecological research offer valuable tools for enhancing various practices and addressing key environmental challenges. Molecular and ecological insights into AMF directly impact agriculture by improving crop yields and resilience. For example, by understanding the genetic and functional traits of AMF, researchers can develop targeted mycorrhizal inoculants that enhance nutrient uptake, particularly phosphorus, and thus boost plant growth in phosphorus-deficient soils. This targeted approach reduces reliance on chemical fertilizers, fostering more sustainable agricultural practices (Khan et al., 2023; Ghorui et al., 2024; Navarro and Morte, 2024).

**Table 3:** Some advances in AMF methodological research.

Methodological advances	Description	References
High-throughput sequencing	Utilization of next-generation sequencing technologies for rapid and comprehensive analysis of AMF diversity and community structure	Hart et al. (2015); Luo et al. (2020); Victcheckorino et al. (2020); Kolaříková et al. (2021); Tchiechoua et al. (2023); Mousa et al. (2024)
Metagenomics	Application of metagenomic approaches to characterize AMF communities and explore their functional potential in various ecosystems	Kang et al. (2020a); Kang et al. (2020b); Shami et al. (2021); Nuccio et al. (2022); Belliardo et al. (2022); Kakouridis et al. (2024); Faghihinia et al. (2024)
Single-nucleus genomics	Development of methods for sequencing individual nuclei to generate *de novo* genome assemblies for AMF species without prior genome data	Lin et al. (2014); Leung et al. (2015); Chen et al. (2018); Masclaux et al. (2019); Chen et al. (2020); Mateus et al. (2022); Lanfranco and Bonfante (2023); Santucciu et al. (2024)
Phylogenomic analyses	Employment of phylogenomic approaches to infer evolutionary relationships among AMF taxa and explore their phylogenetic placement	Corradi et al. (2004); Krüger et al. (2012); Horn et al. (2014); Montoliu-Nerin et al. (1990); Su et al. (2021); Dong et al. (2021); Shu et al. (2021)
Omics techniques	Integration of proteomics, transcriptomics, metatranscriptomics, and metabolomics to gain insights into AMF biology and ecology	Salvioli and Bonfante (2013); Xu et al. (2021); Zhang et al. (2022); Sandrini et al. (2022); Wang et al. (2023b)
Advanced microscopy	Utilization of advanced imaging techniques such as confocal microscopy and electron microscopy for detailed visualization of AMF	Oyarte Galvez et al. (2021); Aparicio Chacón et al. (2023); Hammer et al. (2024)

Recent molecular techniques, such as high-throughput sequencing and functional genomics, have identified specific AMF strains that improve drought resistance and soil structure (Lahlali et al., 2021). Inoculating crops with these beneficial strains can lead to more resilient agricultural systems capable of withstanding environmental stresses like water scarcity and soil degradation (Breuillard et al., 2020). Ecological insights into AMF also play a crucial role in ecosystem restoration and management. AMF contribute significantly to soil health by promoting nutrient cycling and improving soil structure. In degraded ecosystems—such as deforested areas or mined lands—inoculating the soil with AMF can accelerate restoration by enhancing soil fertility and supporting plant re-establishment (Brundrett and Tedersoo, 2018). Moreover, understanding AMF diversity and community dynamics informs biodiversity-friendly land management practices. Preserving native AMF communities helps maintain ecosystem balance and resilience, supporting biodiversity conservation and enhancing ecosystem services like pollination and water regulation (Augé, 2001).

Molecular research on AMF opens up new possibilities for biotechnology. Genetic engineering can be used to modify AMF strains to enhance their beneficial properties, such as stress tolerance or nutrient uptake. Custom strains with optimized traits can be developed for specific agricultural or environmental applications (Reinhart et al., 2024). Additionally, insights into AMF metabolic pathways and gene functions can lead to innovative biofertilizers and biopesticides. Engineering AMF to produce specific compounds that benefit plant health or protect against pathogens offers environmentally friendly alternatives to traditional chemical fertilizers and pesticides (Gianinazzi et al., 2010; Schüßler et al., 2011). Advanced molecular and imaging technologies enable precise monitoring of soil health and AMF dynamics. Techniques like metagenomics and stable isotope probing (SIP) provide detailed insights into microbial community composition and functional activities (Nicol et al., 2008). This data allows soil scientists and farmers to evaluate the effectiveness of AMF inoculants and track changes in soil health over time.

Integrating molecular data with ecological assessments leads to more accurate soil management strategies. For instance, tracking the impact of AMF on soil nutrient levels and microbial diversity helps optimize fertilization practices, crop rotations, and land use, thereby enhancing soil fertility and productivity while minimizing environmental impacts (van der Heijden et al., 2015). The practical applications of molecular and ecological insights into AMF highlight the need for collaborative and interdisciplinary research. Combining expertise from mycology, genomics, ecology, and agronomy leads to comprehensive solutions for global challenges related to food security, environmental sustainability, and ecosystem health (Fitter et al., 2001; Martin, 2024). Such collaborative efforts drive innovation and facilitate the translation of scientific discoveries into practical applications, benefiting both agricultural and natural ecosystems.

### 3.3 Knowledge gaps and research priorities on AMF

The AMF research revealed several critical knowledge gaps that need to be addressed to advance both theoretical and practical applications. Despite significant progress with molecular techniques, gaps persist in the taxonomy and diversity of AMF. Many species remain unidentified, and their distribution across various ecosystems is not fully mapped, impeding a comprehensive understanding of their ecological roles and potential uses (Sudová et al., 2020; Yurkov et al., 2023). To address this, advanced genetic tools such as next-generation sequencing and phylogenetic analysis are essential. These methods can uncover the full diversity of AMF species, their evolutionary relationships, and their ecological niches (Bennett et al., 2013). Understanding the functional diversity of AMF is also crucial. Although AMF species exhibit varied traits related to nutrient acquisition, stress tolerance, and symbiotic efficiency, the implications of these variations for ecosystem processes remain underexplored (Bainard et al., 2017). Research should focus on how different AMF species affect plant performance, soil health, and overall ecosystem function, which is vital for developing sustainable agricultural practices and effective ecosystem management strategies (Martin, 2024).

In addition, exploring the mechanisms of AMF symbiosis with plants represents another priority. While basic mechanisms such as nutrient exchange and mycorrhizal signaling are understood, the detailed molecular pathways and gene regulation processes involved are not fully known (Qi et al., 2022; Shen and Duan, 2024). Investigating these molecular interactions is necessary for enhancing plant growth and stress resilience (Boyno et al., 2023; Gao et al., 2023). Interactions between AMF and environmental factors such as soil conditions, climate change, and pollution also require further study. Soil pH, nutrient availability, and moisture levels significantly affect AMF colonization and function, while climate change and pollution can disrupt AMF communities and their interactions with plants (Rillig et al., 2024; Xiao et al., 2021; Boorboori and Zhang, 2022). Understanding these environmental influences is essential for predicting and mitigating the impacts of global changes on AMF and their ecosystem functions (Crișan et al., 2024).

Research into the dynamics and functional significance of common mycorrhizal networks (CMNs) is also critical. These networks facilitate nutrient and carbon transfer between plants and enhance ecosystem stability, yet their formation, stability, and roles in nutrient and carbon cycling require more investigation (Simard et al., 2012; Selosse et al., 2006). Quantifying the benefits of enhanced mycorrhizal connectivity for different ecosystems and crops can offer valuable insights for improving plant productivity and ecosystem health. The role of AMF in agriculture is well-documented (Tian et al., 2024; Slimani et al., 2024), but the specific impacts of various farming techniques on AMF require further clarification. For example, research has shown that organic farming practices, which often involve reduced use of synthetic chemicals and enhanced soil organic matter, can lead to increased AMF colonization and diversity compared to conventional farming (Wang et al., 2023a). In contrast, intensive tillage has been associated with reduced AMF populations due to soil disruption and decreased fungal spore viability (Kuila and Ghosh, 2022). Additionally, case studies such as those by Tian et al. (2024) demonstrate how implementing cover crops in a no-till system can enhance AMF abundance and soil health by providing continuous organic matter inputs.

The application of AMF in sustainable agriculture, ecosystem restoration, and bioremediation holds significant promise. However, further research is needed to identify the most effective AMF species and inoculation methods for various environments and to assess the long-term impacts of AMF on soil health and ecosystem stability (Kalamulla et al., 2021; Wahab et al., 2023). Long-term field studies are essential for capturing the complexities of AMF’s role in natural ecosystems, as short-term studies may not reflect real-world conditions (Johnson et al., 2010; Brundrett and Tedersoo, 2018). The AMF interact with a diverse range of other soil organisms, including bacteria, fungi, and fauna, which significantly affects plant health and ecosystem functioning (Fall et al., 2022; Hnini et al., 2024). Understanding these complex relationships is crucial for a comprehensive view of soil microbial communities and their roles in ecosystem processes (Wagg et al., 2014). Addressing these knowledge gaps will advance the understanding of AMF biology and its implications for sustainable environmental management.

The advancements in molecular techniques have significantly enhanced understanding of AMF’s ecological roles. For instance, next-generation sequencing technologies have revolutionized our ability to identify AMF species with high resolution, even those that are rare or previously uncharacterized (Bainard et al., 2017; Liu et al., 2014). Metagenomic approaches have provided insights into the functional potential of AMF communities by revealing genes involved in nutrient cycling and stress responses (Wahab et al., 2024; Hyde et al., 2024). A notable example is the previous work, which utilized high-throughput sequencing to uncover previously unknown interactions between AMF and soil bacteria that influence plant health and nutrient uptake (Young and Gillung, 2020).

This review offers several novel insights that differentiate it from existing research. Firstly, the findings highlight a previously unrecognized mechanism by which specific AMF species influence plant resilience to drought, a factor that was not thoroughly addressed in earlier studies (Khaliq et al., 2021). This novel aspect is important as it opens up new avenues for agricultural practices aimed at enhancing crop resilience through targeted AMF inoculation. Additionally, this study demonstrates how integrating molecular data with ecological models can refine predictions about AMF community dynamics in response to environmental changes (Santos-González et al., 2007; Sweeney et al., 2022). Practically, these insights can be applied to develop more effective soil management practices that leverage AMF to improve soil health and crop productivity. For example, this review suggests that tailored AMF inoculants could be used in precision agriculture to optimize nutrient uptake in different soil types. This practical application presents the relevance of the findings to both scientific research and agricultural practice. Existing literature underscores the importance of customizing microbial inoculants, such as AMF and rhizobium, to enhance agricultural productivity and sustainability. Marrassini et al. (2024) reveal that AMF isolates show considerable functional variability depending on soil conditions, with certain isolates promoting better nutrient uptake or plant growth based on soil pH and phosphorus levels. Calderon and Dangi (2024) further demonstrate that the effectiveness of AMF and rhizobium inoculants varies by site, with combinations of AMF and rhizobium improving yields in some dryland conditions and AMF enhancing resilience in acidic soils. Additionally, Paravar et al. (2023) review how microbial seed coatings can enhance seed germination and plant stress tolerance, supporting sustainable agriculture by reducing chemical inputs. Together, these studies highlight the need for tailored inoculant strategies and practices to optimize plant health and productivity across different environments.

## 4 Future directions in AMF research—actionable pathways

The AMF are integral to plant nutrition, soil health, and ecosystem functioning. Despite substantial advancements, significant knowledge gaps and methodological challenges remain, limiting the full utilization of AMF in various applications. This discussion critically assesses these gaps, evaluates past methodologies, and proposes actionable strategies to advance AMF research and applications.

One prominent issue is the taxonomy and diversity of AMF. Although progress has been made, many AMF species remain unidentified, and their distribution across different ecosystems is not fully understood. Traditional morphological methods for identifying AMF are constrained by the high structural similarity among species and an incomplete taxonomic framework. To address these gaps, integrating emerging molecular technologies such as high-throughput sequencing and metagenomics is essential. These technologies offer a comprehensive view of AMF diversity by detecting both known and novel species within complex soil communities (Kalamulla et al., 2021). Conducting global surveys using these advanced methods could map AMF diversity and distribution, leading to the creation of extensive databases that facilitate further research and practical applications.

The functional diversity of AMF is another critical area requiring attention. Although it is acknowledged that AMF species exhibit a range of functional traits, there is insufficient understanding of how these traits vary and their impact on ecosystem processes. Previous research often focused on a limited number of species or traits, missing the broader spectrum of functional diversity within AMF communities (Lee et al., 2013; Albracht et al., 2024). Advanced functional assays and multi-trait analyses utilizing molecular and isotopic techniques can provide insights into how different AMF species contribute to nutrient acquisition, stress tolerance, and soil structure improvement (Wahab et al., 2023). Implementing these studies and integrating ecosystem modeling could predict how variations in AMF functional traits influence nutrient cycling, plant productivity, and soil health, thus informing strategies for optimizing AMF applications in agriculture and conservation (McGale and Sanders, 2022).

Understanding the mechanisms underlying AMF symbiosis with plants is also a significant research priority. While basic mechanisms are known, finer details of the molecular signalling pathways involved in establishing and maintaining symbiotic relationships remain elusive. Research has often concentrated on model species, potentially overlooking the complexity of interactions across diverse plant-AMF pairs (Hu et al., 2022; Hnini et al., 2024). Utilizing transcriptomics and proteomics to map molecular pathways and identify key regulatory genes and proteins is important. Understanding how environmental factors influence these pathways can lead to targeted interventions that enhance symbiosis (Gao et al., 2023).

The AMF interactions with environmental factors such as soil conditions, climate change, and pollution require further exploration. Existing studies often examine these factors in isolation, neglecting the complex interplay between AMF and environmental variables (Rosner and Steinkellner, 2019; Chamard et al., 2024; Kabir et al., 2024). Integrated field and laboratory studies simulating various environmental conditions can provide a more holistic understanding of AMF responses (Feldman et al., 2023; Charakas and Khokhani, 2024; Kozjek et al., 2024). Metagenomic and proteomic approaches to assess adaptation mechanisms can reveal how AMF cope with environmental stresses, which is crucial for developing effective management strategies (Fall et al., 2022).

The dynamics and functional significance of mycorrhizal networks, which connect multiple plants, also warrant deeper investigation. These networks play a vital role in nutrient and carbon cycling, yet their formation, stability, and functions are not fully understood due to past research limitations (Martin, 2024; Ullah et al., 2024). Advanced imaging techniques and network analysis tools can offer insights into the structure and dynamics of mycorrhizal networks (Hammer et al., 2024). Understanding these networks’ roles can help optimize their contribution to ecosystem functions and inform agricultural practices (Hnini et al., 2024).

Applications of AMF in agriculture and ecosystem restoration are increasingly recognized as valuable. Despite their potential benefits, effectively scaling these applications remains a challenge. Long-term field studies are necessary to evaluate the effectiveness of AMF applications in real-world settings (Püschel et al., 2019; Salomon et al., 2021). These studies should focus on the impact of AMF on crop productivity, soil health, and ecosystem restoration, and develop best practices for AMF application based on empirical data. Policy recommendations could support the adoption of AMF-enhanced strategies by providing incentives for farmers and land managers (Gao et al., 2023).

The AMF interactions with a complex array of other soil organisms also merit attention. Previous research may have overlooked these interactions or treated them as secondary to AMF-plant relationships. Holistic approaches that consider the entire soil microbiome, including metagenomic and ecological modeling tools, are needed to understand AMF interactions with other soil microorganisms. Investigating these interactions can reveal their effects on plant health and soil function and inform integrated soil management practices (Kalamulla et al., 2021). Addressing these research priorities and methodological challenges is crucial for advancing the understanding and application of AMF. Leveraging emerging technologies and focusing on actionable research pathways can significantly impact agricultural practices, environmental policies, and ecosystem management. Coordinated efforts among researchers, policymakers, and practitioners will be essential to translate AMF research into tangible benefits for agriculture and conservation.

## 5 Summary of knowledge gaps and research priorities

A significant number of AMF species remain unidentified, which hinders the understanding of their ecological roles and potential applications. Advanced genetic tools, such as next-generation sequencing, are essential for uncovering AMF diversity and elucidating evolutionary relationships. Furthermore, there is a lack of comprehensive understanding regarding how different AMF species influence nutrient acquisition, plant performance, and broader ecosystem processes. Investigating the variations in AMF traits and their impact on ecosystem health and sustainable agricultural practices is a critical research priority.

While basic mechanisms of nutrient exchange and signaling between plants and AMF are known, detailed insights into the molecular pathways and gene regulation processes governing these interactions remain incomplete. Further exploration of these mechanisms could enhance plant growth and resilience to environmental stressors. Additionally, there is a pressing need for research on how soil conditions, climate change, and pollution affect AMF dynamics, as understanding these factors is vital for predicting and mitigating the impacts of global changes on AMF and the ecosystems they inhabit.

Common mycorrhizal networks (CMNs), which play a crucial role in nutrient and carbon transfer between plants, are still under-explored in terms of their formation and specific functions. Quantifying the benefits of these networks is necessary to improve agricultural productivity and ecosystem stability. Although the significance of AMF in agriculture is acknowledged, further investigation is needed into how various farming techniques, such as organic versus conventional practices, affect AMF populations. Long-term studies are essential for accurately assessing AMF’s roles in natural ecosystems, as short-term investigations may not fully reflect real-world conditions.

Moreover, research is required to identify effective AMF species and optimal inoculation methods for sustainable agriculture, ecosystem restoration, and bioremediation efforts. Understanding AMF interactions with other soil organisms is vital for a holistic perspective on soil health. To advance AMF research effectively, future priorities should include the utilization of advanced technologies, such as high-throughput sequencing and metagenomic approaches, which will facilitate mapping AMF diversity and functional potential. Holistic approaches that integrate field and laboratory studies are necessary to comprehend AMF responses to environmental changes thoroughly. Long-term field studies are essential for evaluating AMF applications in real-world settings and developing best practices for their use. Interdisciplinary collaboration among researchers, policymakers, and practitioners will be vital for translating AMF research into practical benefits for agriculture and conservation.

## Data Availability

The original contributions presented in the study are included in the article/supplementary material, further inquiries can be directed to the corresponding author.
